# Serum amino acid profiles and risk of type 2 diabetes among Japanese adults in the Hitachi Health Study

**DOI:** 10.1038/s41598-019-43431-z

**Published:** 2019-05-07

**Authors:** Sanmei Chen, Shamima Akter, Keisuke Kuwahara, Yumi Matsushita, Tohru Nakagawa, Maki Konishi, Toru Honda, Shuichiro Yamamoto, Takeshi Hayashi, Mitsuhiko Noda, Tetsuya Mizoue

**Affiliations:** 1 Department of Epidemiology and Prevention, Center for Clinical Sciences, National Center for Global Health and Medicine, Tokyo, Japan; 2 Department of Clinical Research, Center for Clinical Sciences, National Center for Global Health and Medicine, Tokyo, Japan; 3 Teikyo University Graduate School of Public Health, Tokyo, Japan; 4 Hitachi Health Care Center, Hitachi, Ltd., Ibaraki, Japan; 5 Department of Endocrinology and Diabetes, Saitama Medical University, Saitama, Japan

**Keywords:** Type 2 diabetes, Nutrition, Occupational health, Epidemiology, Risk factors

## Abstract

Amino acids have emerged as novel biomarkers for predicting type 2 diabetes (T2D), but the epidemiologic data linking circulating amino acid profiles with T2D are sparse in Asian populations. We conducted a nested case-control study within a cohort of 4,754 nondiabetic Japanese employees who attended a comprehensive health checkup in 2008–2009 and agreed to provide blood samples. During a 5-year follow-up, incident T2D cases were ascertained based on plasma glucose, glycated hemoglobin, and self-report. Two controls matched to each case on sex, age, and the date of serum sampling were randomly selected by using density sampling, resulting in 284 cases and 560 controls with amino acid measures. High concentrations of valine, leucine, isoleucine, phenylalanine, tyrosine, alanine, glutamate, ornithine, and lysine were associated with an increased risk of incident T2D, in a linear manner. High glutamine concentrations were associated with a decreased risk of incident T2D. Further adjustment for the homeostasis model assessment of insulin resistance attenuated these associations. Overall, these amino acids may be novel useful biomarkers in the identification of people at risk of T2D before overt symptoms. Insulin resistance may account for or mediate the relationship between these amino acids and risk of incident T2D.

## Introduction

Type 2 diabetes (T2D) is a highly prevalent metabolic disorder, characterized by complex disturbances in glucose and lipid metabolism resulting from a combination of resistance to insulin action and an inadequate insulin secretion response^1^. With technological advances in many facets of metabolic profiling, circulating amino acids, which involve insulin action via bidirectional modulation^2,3^, have been suggested to serve as novel biomarkers for predicting the risk of T2D, and perhaps to illuminate previously unknown pathways in diabetes pathophysiology^3,4^.

Circulating branched-chain amino acids (BCAAs; valine, leucine, and isoleucine) and aromatic amino acids (phenylalanine and tyrosine) have consistently shown positive associations with the risk of developing T2D^4–12^. Evidence regarding other amino acids is inconsistent. For instance, an inverse association of glutamine with the risk of T2D has been observed in some^10,12,13^, but not all^5,6,8,11^, studies. Glutamate was positively associated with incident T2D in cohort studies of Finnish^12^ and American adults^13^, whilst showing no association in a cohort of Swedish subjects^13^. Glycine was found to be inversely associated with incident T2D in Germany and Finland^6,11,12^, but no association was observed among South Asians in the United Kingdom^8^. Alanine and histidine, as well as ornithine, were also positively associated with T2D, although evidence is limited^4^.

Most prior studies on this issue have been performed in European and American populations, whilst evidence in Asian populations is sparse. A prospective study in China examined three BCAAs and two aromatic amino acids in association with T2D^9^. A prospective study in Japanese adults reported associations of a composite amino acid score of 19 amino acids with diabetes^14^. Ethnic differences in patterns of associations between amino acids and T2D have been identified between Europeans and South Asian Europeans^8^. Provided Westerners’ and Eastern Asians’ interethnic differences in interaction between genetic, pathophysiological, cultural, and lifestyle factors related to the development of T2D^15^, amino acid profiles may show a distinct pattern of associations with T2D in Japanese populations. Here, we carried out a case-control study nested within a Japanese cohort, to examine associations between circulating amino acids and the risk of T2D.

## Results

**Figure 1:**
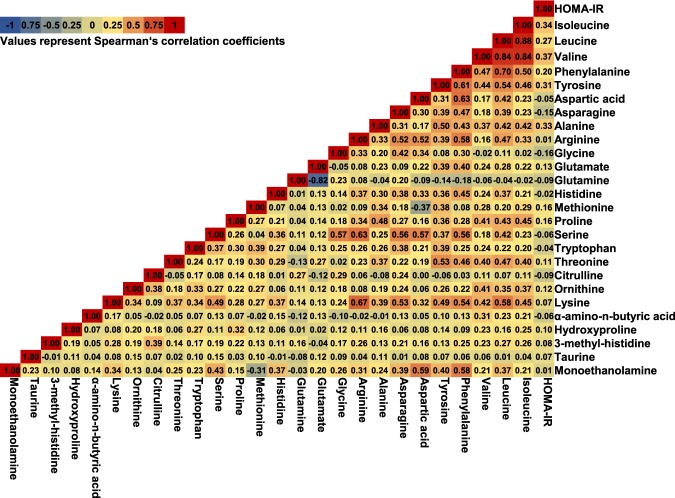
The correlations among baseline amino acids and HOMA-IR in control subjects in the Hitachi Health Study.

Baseline characteristics are summarized in Table 1. Compared with control subjects, case subjects had higher levels of body mass index (BMI), glucose, glycated hemoglobin (HbA1c), insulin, and homeostasis model assessment of insulin resistance (HOMA-IR), and were more likely to have hypertension and family history of diabetes. The two groups had no significant difference in terms of smoking, drinking status, sleep duration, shift work, or occupational and leisure-time physical activity level. The correlations among baseline amino acids and HOMA-IR in control subjects are illustrated in Fig. 1. Three BCAAs (isoleucine, leucine, and valine) were clustered into subgroups with strong correlations (ρ = 0.84–0.88), as well as glutamine and glutamate (ρ = −0.82). Other amino acids were moderately or weakly correlated with each other except for a strong correlation between phenylalanine and leucine (ρ = 0.70). HOMA-IR significantly correlated with 10 of these 26 amino acid metabolites. The highest correlation coefficients with HOMA-IR were found for BCAAs and aromatic amino acids (the highest ρ = 0.37 for valine), followed by alanine, asparagine, methionine, proline, and glycine (all P < 0.002).

**Table 1:** Baseline characteristics of diabetic case subjects and control subjects.

Characteristics	Cases (n = 284)	Controls (n = 560)	P value^a^
Age, mean ± SD, years	51.2 ± 7.3	50.8 ± 7.3	0.58
Male, %	89.9	90.0	0.92
Smoking status, %			0.51
Never smoker	35.2	34.6	
Former smoker	27.1	31.4	
Current smoker consuming <20 cigars/d	5.3	3.9	
Current smoker consuming ≥20 cigars/d	32.4	30	
Drinking status, %			0.66
Non-drinker	23.9	24.8	
Alcohol consuming <23 g ethanol/day	39.4	40.3	
Alcohol consuming 23–<46 g ethanol/d	22.2	23.4	
Alcohol consuming ≥46 g ethanol/d	14.4	11.4	
Sleeping duration, %			0.92
<6 hours/day	48.0	47.9	
6 to <7 hours/day	42.7	41.9	
≥7 hours/day	9.9	9.5	
Shift work, %	12.0	10.7	0.58
Occupational physical activity **(**sedentary), %	62.3	64.3	0.58
Leisure time physical activity (≥150 mins/week), %	19.4	15.9	0.20
Family history of type 2 diabetes, %	25.7	19.3	0.03
Hypertension, %	29.2	17.1	<0.001
BMI, kg/m^2^	25.3 ± 3.4	23.4 ± 2.6	<0.001
Fasting glucose, median (IQR), mmol/L	113 (106–117)	99 (95–105)	<0.001
HbA1c, % (mmol/mol)	6.0 ± 0.3	5.7 ± 0.3	<0.001
Fasting insulin, median (IQR), μU/mL	6.6 (4.3–10.5)	5.0 (3.3–7.1)	<0.001
HOMA-IR, median (IQR)	1.8 (1.2–3.0)	1.2 (0.8–1.9)	<0.001

Abbreviations: SD, standard deviation; BMI, body mass index; IQR, interquartile range; HbA1c, glycated hemoglobin; HOMA-IR, homeostasis model assessment of insulin resistance.

^a^Comparisons between cases and controls using chi-square test for categorical variables, *t*-test for normally distributed continuous variables, and Mann-Whitney’s *U* test for skewed continuous variables.

Of the 26 amino acids, 10 were identified to be significantly associated with risk of incident T2D with adjustment for age, sex, month of health check-up, leisure time physical activity, occupational physical activity, smoking, alcohol consumption, shift work, sleep duration, family history of diabetes, hypertension, and BMI. As shown in Table 2 (model 2), individuals in the top quartiles of concentrations of BCAAs (isoleucine, leucine, and valine) and aromatic amino acids (phenylalanine and tyrosine) had 1.7- to 2.6-fold higher odds of newly developed T2D, compared with individuals in the lowest quartile (all P for trend <0.05). Additional adjustment for HOMA-IR abolished the statistical significances of the associations of valine, isoleucine, and tyrosine with incident T2D (Table 2; model 3).

**Table 2:** Associations of serum concentrations of BCAAs and aromatic amino acids with incident type 2 diabetes in the Hitachi Health Study.

	Quartiles (range)^a^ (μmol/L)	No. of ca./co.	Model 1 OR (95% CI)	Model 2 OR (95% CI)	Model 3 OR (95% CI)
Isoleucine	1 (<69.4)	36/137	1.00	1.00	1.00
	2 (69.4–<80.3)	61/142	2.09 (1.25–3.50)	1.82 (1.05–3.13)	1.47 (0.81–2.68)
	3 (80.3–<90.1)	88/142	3.25 (1.93–5.48)	2.51 (1.44–4.44)	1.97 (1.09–3.55)
	4 (≥90.1)	99/139	3.78 (2.25–6.35)	2.53 (1.43–4.49)	1.68 (0.91–3.10)
	P-trend^b^		<0.001	0.002	0.13
Leucine	1 (<146.2)	37/140	1.00	1.00	1.00
	2 (146.2–<161.2)	52/140	1.68 (1.01–2.79)	1.40 (0.82–2.41)	1.21 (0.66–2.19)
	3 (161.2–<178.8)	86/139	2.95 (1.80–4.85)	2.38 (1.40–4.05)	2.03 (1.14–3.61)
	4 (≥178.8)	109/141	3.72 (2.28–6.07)	2.60 (1.53–4.41)	2.06 (1.16–3.67)
	P-trend^b^		<0.001	<0.001	0.005
Valine	1 (<239.1)	38/140	1.00	1.00	1.00
	2 (239.1–<263.8)	63/140	2.00 (1.22–3.29)	1.59 (0.94–2.67)	1.28 (0.73–2.26)
	3 (263.8–<290.8)	74/142	2.42 (1.47–4.00)	1.71 (1.01–2.91)	1.26 (0.71–2.23)
	4 (≥290.8)	109/138	3.69 (2.26–6.02)	2.34 (1.38–3.96)	1.48 (0.84–2.61)
	P-trend^b^		<0.001	0.002	0.20
Phenylalanine	1 (<82.8)	37/141	1.00	1.00	1.00
	2 (82.8–<91.1)	71/144	1.86 (1.17–2.95)	1.62 (0.99–2.65)	1.48 (0.87–2.52)
	3 (91.1–<101.2)	76/139	2.08 (1.31–3.31)	1.67 (1.02–2.73)	1.47 (0.86–2.50)
	4 (≥101.2)	100/140	2.72 (1.73–4.27)	2.10 (1.29–3.42)	1.76 (1.04–2.97)
	P-trend^b^		<0.001	0.005	0.06
Tyrosine	1 (<64.7)	50/140	1.00	1.00	1.00
	2 (64.7–<72.0)	43/142	0.86 (0.54–1.38)	0.70 (0.42–1.15)	0.58 (0.33–1.00)
	3 (72.0–<79.8)	53/136	1.16 (0.73–1.83)	0.86 (0.53–1.41)	0.65 (0.038–1.09)
	4 (≥79.8)	138/142	2.91 (1.93–4.39)	1.72 (1.08–2.71)	1.20 (0.73–1.98)
	P-trend^b^		<0.001	0.002	0.10

Abbreviations: OR, odds ratio; CI, confidential interval; BCAAs, branched-chain amino acid; BMI, body mass index; HOMA-IR, homeostasis model assessment of insulin resistance.

Model 1 was adjusted for the following matching factors: age (years), sex, and month of health examination (April-June, July-Sep, Oct-Dec, or Jan-March). Model 2 was based on model 1, additionally adjusted for leisure time physical activity (<150 minutes/week or ≥150 minutes/week), occupational physical activity (sedentary or active), smoking (never smoker, former smoker, or current smoker consuming <20 or ≥20 cigarettes/day), alcohol consumption (non-drinker, or alcohol consuming <23, 23 to <46, or ≥46 g ethanol/day), shift work (yes or no), sleep duration (<6, 6 to <7, or ≥7 hours/day), family history of diabetes (yes or no), and hypertension (yes or no), and BMI. Model 3 was further adjusted for HOMA-IR.

^a^Quartiles were based on the distribution of serum concentrations among controls.

^b^All linear trend tests over quartiles were conducted by replacing the ordinal values with the median concentration within each quartile.

With regard to gluconeogenic amino acids and other amino acids (Table 3), alanine, glutamate, ornithine, and lysine concentrations displayed positive associations with incident T2D (model 2; adjusted odds ratios [ORs] 1.61 to 2.91 for the highest quartile, compared with the lowest quartile, all P < 0.05), in a linear manner (all P for trend <0.01). Glutamine (adjusted ORs [95% confidence intervals, CI] 0.57 [0.35–0.93] for the highest quartile, compared with the lowest quartile; P for trend = 0.04) and the glutamine-to-glutamate ratio (adjusted ORs [95% CI] 0.36 [0.21–0.62] for the highest quartile, compared with the lowest quartile; P for trend <0.001) were inversely associated with risk of incident T2D. After adjustment for HOMA-IR, the associations of alanine, ornithine, and lysine concentrations were attenuated and became statistically not significant (Table 3; model 3). The associations for glutamate and the glutamine-to-glutamate ratio were attenuated but remained statistically significant. With respect to the remaining five intermediary organic acids (Supplemental Table S1), none were significant predictors of T2D (P > 0.05 for all models).

**Table 3:** Associations of serum concentrations of gluconeogenic amino acids and other amino acids with incident type 2 diabetes in the Hitachi Health Study.

	Quartiles (range)^a^ (μmol/L)	No. of ca./co.	Model 1 OR (95% CI)	Model 2 OR (95% CI)	Model 3 OR (95% CI)
Aspartic acid	1 (<35)	64/149	1.00	1.00	1.00
	2 (35–<40)	64/132	1.13 (0.74–1.72)	1.16 (0.74–1.83)	1.16 (0.72–1.87)
	3 (40–<47)	77/141	1.28 (0.85–1.92)	1.36 (0.88–2.12)	1.42 (0.89–2.27)
	4 (≥47)	79/138	1.35 (0.89–2.04)	1.49 (0.95–2.32)	1.45 (0.90–2.34)
	P-trend^b^		0.14	0.07	0.10
Asparagine	1 (<45.4)	90/140	1.00	1.00	1.00
	2 (45.4–<51.4	66/138	0.74 (0.50–1.10)	0.89 (0.58–1.36)	0.94 (0.59–1.50)
	3 (51.4–<58.0)	57/142	0.61 (0.41–0.93)	0.76 (0.49–1.18)	0.94 (0.59–1.51)
	4 (≥58.0)	71/140	0.78 (0.53–1.16)	0.95 (0.62–1.45)	1.15 (0.73–1.83)
	P-trend^b^		0.20	0.75	0.52
Alanine	1 (<409.4)	45/140	1.00	1.00	1.00
	2 (409.4–<461.5)	48/140	1.12 (0.69–1.80)	0.93 (0.59–1.61)	0.80 (0.46–1.36)
	3 (461.5–<528.4)	87/140	2.03 (1.31–3.16)	1.52 (0.95–2.44)	1.19 (0.72–1.98)
	4 (≥528.4)	104/140	2.43 (1.58–3.75)	1.61 (1.01–2.57)	0.98 (0.59–1.64)
	P-trend^b^		<0.001	0.02	0.73
Arginine	1 (<149.2)	65/141	1.00	1.00	1.00
	2 (149.2–<170.6)	79/139	1.23 (0.82–1.84)	1.35 (0.88–2.10)	1.32 (0.83–2.11)
	3 (170.6–<205.6)	64/140	0.99 (0.65–1.50)	1.13 (0.72–1.78)	1.14 (0.70–1.85)
	4 (≥205.6)	76/140	1.17 (0.78–1.77)	1.23 (0.79–1.91)	1.30 (0.81–2.07)
	P-trend^b^		0.66	0.64	0.46
Glycine	1 (<268.9)	79/140	1.00	1.00	1.00
	2 (268.9–<299.7)	74/140	0.94 (0.63–1.39)	1.00 (0.65–1.54)	1.12 (0.71–1.76)
	3 (299.7–<334.9)	62/140	0.79 (0.52–1.18)	1.04 (0.67–1.62)	1.25 (0.78–2.01)
	4 (≥334.9)^b^	69/140	0.87 (0.58–1.30)	1.28 (0.82–1.99)	1.63 (0.99–2.63)
	P-trend		0.42	0.26	0.04
Glutamate	1 (<208.6)^b^	33/140	1.00	1.00	1.00
	2 (208.6–<260.9)	58/140	2.05 (1.23–3.43)	1.85 (1.08–3.18)	1.70 (0.94–3.07)
	3 (260.9–<337.6)	84/139	3.10 (1.88–5.12)	2.24 (1.30–3.86)	1.84 (1.02–3.34)
	4 (≥337.6)	109/141	4.09 (2.48–6.74)	2.91 (1.67–5.08)	2.33 (1.27–4.25)
	P-trend^b^		<0.001	0.001	0.02
Glutamine	1 (<296.2)	108/139	1.00	1.00	1.00
	2 (296.2–<362.9)	57/141	0.51 (0.34–0.77)	0.60 (0.38–0.93)	0.55 (0.34–0.88)
	3 (362.9–<420.0)	69/140	0.62 (0.42–0.91)	0.79 (0.51–1.22)	0.98 (0.62–1.55)
	4 (≥420.0)	50/140	0.44 (0.29–0.67)	0.57 (0.35–0.93)	0.72 (0.42–1.20)
	P-trend^b^		<0.001	0.04	0.30
Histidine	1 (<92.9)	63/140	1.00	1.00	1.00
	2 (92.9–<100.4)	65/138	1.06 (0.69–1.62)	0.94 (0.59–1.48)	0.87 (0.53–1.43)
	3 (100.4–<108.6)	69/142	1.11 (0.73–1.69)	1.02 (0.65–1.61)	0.92 (0.57–1.50)
	4 (≥108.6)	87/140	1.40 (0.93–2.11)	1.25 (0.81–1.92)	1.26 (0.79–2.00)
	P-trend^b^		0.09	0.23	0.21
Methionine	1 (<16.8)	60/140	1.00	1.00	1.00
	2 (16.8–<20.8)	72/140	1.27 (0.82–1.95)	1.17 (0.74–1.85)	1.20 (0.73–1.98)
	3 (20.8–<24.2)	62/138	1.13 (0.72–1.77)	0.91 (0.56–1.49)	0.97 (0.58–1.64)
	4 (≥24.2)	90/142	1.62 (1.05–2.52)	1.18 (0.73–1.89)	1.18 (0.72–1.94)
	P-trend^b^		0.04	0.66	0.68
Proline	1 (<151.2)	52/140	1.00	1.00	1.00
	2 (151.2–<177.0)	81/141	1.60 (1.04–2.47)	1.28 (0.81–2.03)	1.27 (0.77–2.09)
	3 (177.0–<209.5)	67/139	1.37 (0.88–2.14)	1.08 (0.67–1.74)	0.90 (0.53–1.49)
	4 (≥209.5)	84/140	1.72 (1.11–2.66)	1.28 (0.80–2.06)	1.08 (0.65–1.78)
	P-trend^b^		0.05	0.47	0.89
Serine	1 (<154.4)	68/140	1.00	1.00	1.00
	2 (154.4–<168.8)	64/136	0.96 (0.63–1.46)	0.91 (0.58–1.42)	0.76 (0.47–1.21)
	3 (168.8–<187.1)	67/141	0.98 (0.65–1.48)	0.95 (0.61–1.48)	0.98 (0.61–1.57)
	4 (≥187.1)	85/143	1.23 (0.83–1.84)	1.30 (0.85–1.99)	1.42 (0.90–2.24)
	P-trend^b^		0.25	0.16	0.05
Tryptophan	1 (<56.2)	57/139	1.00	1.00	1.00
	2 (56.2–<61.2)	60/141	1.08 (0.70–1.68)	0.98 (0.61–1.56)	0.92 (0.55–1.52)
	3 (61.2–<66.3)	61/140	1.13 (0.72–1.76)	0.85 (0.53–1.37)	0.74 (0.44–1.24)
	4 (≥66.3)	106/140	2.01 (1.32–3.07)	1.46 (0.92–2.31)	1.38 (0.84–2.24)
	P-trend^b^		0.001	0.09	0.15
Threonine	1 (<128.5)	70/140	1.00	1.00	1.00
	2 (128.5–<145.9)	71/140	1.02 (0.68–1.53)	0.87 (0.56–1.35)	0.92 (0.57–1.48)
	3 (145.9–<163.6)	67/140	0.96 (0.64–1.45)	0.81 (0.52–1.27)	0.92 (0.57–1.48)
	4 (≥163.6)	76/140	1.09 (0.73–1.64)	0.98 (0.63–1.52)	1.15 (0.72–1.84)
	P-trend^b^		0.72	0.94	0.51
Citrulline	1 (<26.0)	80/145	1.00	1.00	1.00
	2 (26.0–<29.8)	80/142	1.01 (0.68–1.50)	1.12 (0.73–1.72)	1.18 (0.75–1.86)
	3 (29.8–<33.4)	51/132	0.69 (0.45–1.06)	0.87 (0.55–1.38)	0.89 (0.54–1.46)
	4 (≥33.4)	73/141	0.92 (0.62–1.38)	1.22 (0.78–1.90)	1.31 (0.82–2.11)
	P-trend^b^		0.45	0.53	0.38
Ornithine	1 (<55.1)	53/140	1.00	1.00	1.00
	2 (55.1–<62.7)	55/139	1.07 (0.68–1.68)	1.03 (0.64–1.66)	0.97 (0.58–1.64)
	3 (62.7–<71.2)	83/141	1.60 (1.04–2.46)	1.45 (0.92–2.29)	1.29 (0.80–2.11)
	4 (≥71.2)	93/140	1.82 (1.19–2.79)	1.72 (1.09–2.72)	1.57 (0.96–2.56)
	P-trend^b^		0.001	0.007	0.03
Lysine	1 (<205.5)	53/140	1.00	1.00	1.00
	2 (205.5–<224.7)	60/143	1.13 (0.72–1.76)	0.98 (0.60–1.59)	0.86 (0.51–1.44)
	3 (224.7–<250.0)	69/137	1.36 (0.88–2.10)	1.16 (0.73–1.86)	1.06 (0.64–1.75)
	4 (≥250.0)	102/140	1.98 (1.30–3.02)	1.63 (1.03–2.57)	1.47 (0.91–2.38)
	P-trend^b^		<0.001	0.01	0.03

Abbreviations: OR, odds ratio; CI, confidential interval; BMI, body mass index; HOMA-IR, homeostasis model assessment of insulin resistance. Model 1 was adjusted for the following matching factors: age (years), sex, and month of health examination (April-June, July-Sep, Oct-Dec, or Jan-March). Model 2 was based on model 1, additionally adjusted for leisure time physical activity (<150 minutes/week or ≥150 minutes/week), occupational physical activity (sedentary or active), smoking (never smoker, former smoker, or current smoker consuming <20 or ≥20 cigarettes/day), alcohol consumption (non-drinker, or alcohol consuming <23, 23 to <46, or ≥46 g ethanol/day), shift work (yes or no), sleep duration (<6, 6 to <7, or ≥7 hours/day), family history of diabetes (yes or no), and hypertension (yes or no), and BMI. Model 3 was further adjusted for HOMA-IR.

^a^Quartiles were based on the distribution of serum concentrations among controls.

^b^All linear trend tests over quartiles were conducted by replacing the ordinal values with the median concentration within each quartile.

Sensitivity analyses using conditional logistic analyses showed a virtually similar pattern of associations between amino acid profiles and the risk of T2D.

## Discussion

In this prospective nested case-control study among Japanese working adults, we demonstrated that fasting serum concentrations of several amino acids, including BCAAs (valine, leucine, and isoleucine), aromatic amino acids (phenylalanine and tyrosine), gluconeogenic amino acids (alanine, glutamate, and glutamine), and other amino acids (ornithine and lysine), were associated with the risk of T2D. After further adjustment for HOMA-IR, most of these associations were largely attenuated and became no longer statistically significant, except for leucine, phenylalanine, glutamate, and the glutamine-to-glutamate ratio, whose associations were attenuated but remained statistically significant.

The positive associations of BCAAs and aromatic amino acids with the risk of T2D in the current study among Japanese are in line with those reported among Western populations^5–8,10–12^. Consistently, a meta-analysis of eight cohort studies concluded that BCAAs and aromatic amino acids are significantly associated with the incidence of T2D^4^. Our findings are also comparable to the limited evidence in Asia. In a nested case-control study of 429 Chinese adults, serum BCAAs and aromatic amino acids were significantly and positively associated with T2D^9^. In a large-scale cross-sectional study among Japanese, individuals with diabetes had higher levels of BCAAs and aromatic amino acids than those who were non-diabetic^16^. Hence, our findings not only confirmed previous findings in Western populations but also extended the evidence in Asia, supporting the notion that circulating BCAAs and aromatic amino acids may potentially contribute to the risk of developing T2D.

We found that an elevated level of alanine was associated with increased risk of T2D among the Japanese population, in line with the results reported among Finnish^10,12^, American^7^, and South Asian men in the United Kingdom^8^. Alanine can be synthesized from pyruvate by the glucose-alanine cycle^17^. Elevated alanine aminotransferase, a key enzyme of the glucose-alanine cycle in which alanine is formed, has been associated with a higher risk of developing T2D^18,19^, concordant with our findings. The causality between alanine and the risk of T2D is still to be determined; however, our findings highlight attention to a likely role for alanine in the development of T2D.

Existing evidence on the associations of glutamine and glutamate with incident T2D is conflicting. We found that glutamine was inversely associated with incident T2D in Japanese. Some of the previous cohort studies have also reported that a high glutamine level is associated with a lower risk of T2D^10,12,13^, but others reported no association^5,6,8,11^. We found a positive association of glutamate with the risk of T2D, consistent with that reported in a previous cohort study^12^. In the Framingham Heart Study, Cheng *et al*.^13^ observed a positive association of glutamate, but they failed to find a significant association in the replication cohort (Malmö Diet and Cancer Study). The results from the Framingham Offspring Study did not show such an association for glutamate either^5^. In mice, excess glutamine relative to glutamate, resulting from the exogenous administration, has been shown to improve metabolic profiles^13^, favoring the associations of glutamine and glutamate with incident T2D. Our results confirmed a positive association between high glutamine-to-glutamate ratio and risk of T2D. Taken together, despite those inconsistencies in previous cohort studies among Western populations, our findings from a Japanese population further corroborated the links between circulating glutamine, glutamate, and the risk of T2D.

Fish intake, the main dietary source of glycine for Japanese^20^, has been found to be associated with a lower risk of T2D^21^. In the present study, however, we found no significant association of circulating glycine with the risk of T2D. This finding agrees with results from a cohort study in the United Kingdom, which reported that glycine was not associated with incident T2D in South Asians^8^. An inverse association of glycine with T2D has been reported in previous cohort studies of Europeans^6,11,12^, whereas a Mendelian randomization analysis in a European population showed no association between genetic variants associated with glycine and T2D, concordant with our observation of a null association between circulating glycine and T2D. Therefore, we postulate that circulating glycine, even at higher levels, may not influence T2D risk.

Although the associations of ornithine and lysine with the risk of T2D were not significant in Western studies^6,7,11,12^, we found positive associations of lysine and ornithine with the risk of T2D. Our findings are concordant with results from a cross-sectional study among Japanese adults, which showed an association of elevated ornithine and lysine with a higher likelihood of being diabetic^16^. The concentration of lysine, an essential amino acid mainly obtained from diet, was higher in the present study population than in a Finnish^12^ and a German^11^ study population (mean lysine: 225 μmol/L vs. 157 and 166 μmol/L), which may result in the difference in the association for lysine. Ornithine is the product of the urea cycle. Increased arginase activity, a key enzyme for creating ornithine from arginine in the urea cycle, has been found in T2D subjects^22^, in agreement with our observation of elevated ornithine associated with T2D. Given the paucity of evidence on these associations for lysine and ornithine, additional studies are warranted to confirm our observations.

Insulin resistance plays a crucial role in the pathogenesis of T2D, leading to T2D susceptibility. In the present study, we demonstrated that adjustment for insulin sensitivity attenuated most of the associations. Previous studies have also reported that additional adjustment for insulin resistance attenuated the relationship between amino acid levels and the risk of diabetes^7,8,10^. One possible explanation is that insulin resistance causes increased levels of circulating amino acids; thus, the observed associations between amino acids and T2D may be confounded by insulin resistance. In support of this explanation, a large-scale Mendelian randomization analysis provided genetic evidence that high levels of insulin resistance lead to an elevation in circulating branched-chain amino acids^23^. Another possible explanation is that insulin resistance mediates the associations between amino acids and T2D; that is, amino acids increase or decrease the risk of T2D through its effect on insulin resistance. Circulating amino acids have been shown to be predictive of the development of insulin resistance^7,24,25^, and promote insulin resistance via inhibiting insulin signalling^2,26,27^. Glutamine administration increases insulin sensitivity^28^ and may consequently decrease the risk of T2D. The associations for leucine, phenylalanine, glutamate, and the glutamine-to-glutamate ratio remained significant after adjustment for insulin resistance, suggesting that there might be some mechanisms, other than insulin resistance, behind those associations. For instance, amino acids, such as leucine, phenylalanine, glutamine, and glutamate, induce pancreatic β-cell insulin secretion^27,29–31^ and may promote T2D via hyperinsulinemia, leading to pancreatic β-cell exhaustion^5^. Some other mechanisms such as stimulating glucagon release from pancreatic α- cells^32^ and increasing transamination of pyruvate to alanine, a strong promoter of gluconeogenesis^26^, may explain the potentially unfavorable role of glutamate on T2D risk. Despite unclear mechanisms, those amino acids may be novel useful biomarkers of T2D^33^.

Strengths of this study include the prospective design nested in a well-characterized cohort, the annual ascertainment of diabetes based on data from health check-ups, and the blinding to case-control status when analyzing amino acid concentrations. Limitations should also be considered. First, we did not use the oral glucose tolerance test for the diagnosis of diabetes. However, the International Expert Committee has recommended the HbA1c assay in the diagnosis of diabetes^34^. The ease of sampling collection for the HbA1c test supports using HbA1c to diagnose diabetes in epidemiological studies^34^. Second, we only targeted a set of major amino acids. Expanding the coverage of amino acid metabolites will facilitate identification of other new biomarkers for T2D. Third, amino acid profiles in serum might be less stable than profiles in plasma, but serum samples provide more sensitive results in amino acid detection^35,36^. Fourth, we measured amino acid profiles only at baseline. This might have caused exposure misclassification in a random manner, resulting in an attenuation of the association between amino acid concentrations and the risk of T2D. Finally, the data were derived from a cohort of Japanese working adults. Caution should be exercised when generalizing our findings to other populations.

In summary, this nested case-control study among Japanese adults demonstrated that serum concentrations of valine, leucine, isoleucine, phenylalanine, tyrosine, alanine, glutamine, glutamate, ornithine, and lysine are associated with the risk of incident T2D, mediated or confounded by insulin resistance. These amino acids may be novel useful biomarkers in the identification of people at risk of T2D before overt symptoms, and potential targets for illuminating previously unknown pathways in T2D pathophysiology.

## Methods

### Study design

The Hitachi Health Study is an ongoing prospective cohort study including baseline subjects comprising 17,606 employees and their spouses, and retired employees who participated in an annual comprehensive health check-up at Hitachi Health Care Center (Hitachi, Japan) between April 2008 and March 2009^37^. Of those, 7,993 agreed to respond to a study questionnaire and provide blood samples. Peripheral venous blood samples were collected after overnight fasting and stored in freezers at −80 °C until analysis.

The flow diagram of sampling for this present study is shown in Supplemental Fig. S1. Exclusion criteria for this present study included a history of cancer, stroke, or myocardial infarction at baseline; a history of diabetes or having no measures of neither fasting glucose nor HbA1c at baseline; absence from all subsequent annual health check-ups during follow-up; and retired employees or their spouses. Accordingly, 4,754 employees aged 34 to 69 years were eligible for the biomarker cohort study, in which we carried out a nested case-control study of T2D. All participants were followed up on a basis of annual health check-up until March 2014.

As previously described^38,39^, we identified a total of 357 new-onset T2D cases over a 5-year follow-up period. We randomly selected two controls for each case by using incidence density sampling, which involves matching each case to each risk set from cohort subjects under follow-up at the time of case occurrence^40^. Cases and controls were matched for sex, age at serum sampling (≤2 years), and the date of serum sampling (≤2 weeks). Selected control of one case could later become a case, and a case was allowed to be used again as the control of other cases. We excluded 9 cases due to failure to find a matched control or having no or too small volume of blood sample, resulting in 348 cases and their matched 694 controls. Of these, 64 cases and 134 controls had an insufficient volume of serum left for amino acid measurements. In the remaining 844 subjects, 12 cases having no remaining controls and 94 controls without remaining cases were not excluded from the final sample in order to maximize statistical power. Thus, the final sample comprised 284 cases and 560 controls.

All methods were performed in accordance with the relevant institutional and national guidelines and regulations. The study protocol was approved by the Ethics Review Committee of the National Center for Global Health and Medicine, Japan. Written informed consent was obtained from all participants.

### Amino acid profiles measurements

Serum levels of amino acids were measured by high-performance liquid chromatography-mass spectrometry (HPLC). Analyses were performed on the 2000 QTRAP ACQUITY UPLC (MS) system with an ODS C18 column (2.1 × 100 mm) by using the MassTrak™ Amino Acid Analysis Solution kit (Waters Corp., Tokyo, Japan). We initially targeted 39 amino acid metabolites. Of these, 26 were detectable and eligible for final analyses, namely, three BCAAs (isoleucine, leucine, and valine), two aromatic amino acids (phenylalanine, tyrosine), 16 gluconeogenic amino acids and other amino acids (aspartic acid, asparagine, alanine, arginine, glycine, glutamate, glutamine, histidine, methionine, proline, serine, tryptophan, threonine, citrulline, ornithine, and lysine), and five intermediary organic acids (α-amino-n-butyric acid, hydroxyproline, 3-methylhistidine, taurine, and monoethanolamine). We also calculated a metabolite ratio of glutamine-to-glutamate. Concentrations of these metabolites were expressed in micromoles (μmol) per liter (L).

### Ascertainment of T2D

The onset of T2D was identified using data from subsequent annual health check-ups. According to the American Diabetes Association criteria^41^, diabetes during the follow-up period was defined as presentation of at least one of the following criteria: (1) HbA1c ≥6.5% (48 mmol/L), (2) fasting glucose ≥126 mg/dL (7.0 mmol/L), (3) random plasma glucose ≥200 mg/dL (11.1 mmol/L); or (4) currently under medical treatment for T2D.

### Other variables

Baseline data for all health-related lifestyle factors, including smoking, alcohol consumption, sleep duration, shift work, occupational and leisure time physical activity, medical history and family history of chronic diseases, and medication use, were collected at the time of blood sampling via a questionnaire. Weight and height were measured based on standard procedures using an automated scale (BF-220, Tanita, Tokyo, Japan). BMI was computed as weight/height^2^ (kg/m^2^). Blood pressure (mmHg) was assessed with an automated sphygmomanometer (BP203RV; Colin). Hypertension was defined as systolic blood pressure ≥140 mmHg, diastolic blood pressure ≥90 mmHg, or taking anti-hypertensive medications. Plasma glucose (mg/dL) was analyzed enzymatically (A&T, Tokyo, Japan) and serum insulin (μU/mL) immunoenzymatically (Abbott, Tokyo, Japan). The index of insulin resistance, HOMA-IR, was calculated using the following formula: (fasting serum insulin [μU/mL] × fasting plasma glucose [mg/dL])/405. HbA1c (Japan Diabetes Society, %) was measured using the HPLC method (HLC723-G9, TOSOH, Tokyo, Japan), and then converted into a National Glycohemoglobin Standardization Program equivalent value (%) by adding 0.4% to the measured value^42^.

### Statistical analyses

Baseline characteristics of incident diabetes case subjects and matched control subjects were compared using chi-square test for categorical variables, *t*-test for normally distributed continuous variables, and Mann-Whitney’s *U* test for skewed continuous variables. Multiple imputations were used to handle missing data on covariates’ shift work (n = 9), occupational physical activity (n = 12), and leisure time physical activity (n = 1). We performed multiple imputations with 10 iterations and combined the estimates from each imputed data set^43^. We calculated Spearman’s rank correlation coefficients (ρ) across baseline concentrations of amino acids with HOMA-IR among control subjects.

We categorized all subjects according to the quartile cut-off of each amino acid among controls. For each amino acid (predictor variable), unconditional logistic regression analyses were run to estimate ORs and 95% CIs for the risk of T2D onset with the lowest quartile as the reference. The choice of unconditional analysis for case-control studies has been recommended because it produces better statistical precision than matched analyses^44^. We built a minimally adjusted model (model 1) including age (years), sex, and month of health check-up (April-June, July-September, October-December, or January-March). We then built the second model adjusted for all covariates (model 2), additionally including smoking (never smoker, former smoker, or current smoker consuming <20, or ≥20 cigarettes/day), alcohol consumption (non-drinker, or alcohol consuming <23, 23 to <46, or ≥46 g ethanol/day), sleep duration <6, 6 to <7, or ≥7 hours/day), shift work (yes or no), occupational physical activity (sedentary or active), leisure time physical activity (<150 minutes/week or ≥150 minutes/week), family history of diabetes (yes or no), hypertension (systolic/diastolic blood pressure ≥140/90 mmHg or anti-hypertensive medication use), and BMI at baseline. To test the effects of HOMA-IR on associations between amino acids and incident T2D, we built the third model with additional adjustment for baseline HOMA-IR (log transformed). Trend association was assessed by assigning the median value to each quartile category of each amino acid and entering this variable into the model as a continuous variable.

We performed sensitivity analyses of replicating analyses of assessing associations between amino acid profiles and incident T2D by using conditional logistic models, adjusting for all covariates with and without HOMA-IR. To account for the multiple testing of correlations between 26 amino acid metabolites and HOMA-IR, we used a corrected P value threshold of 0.002 (=0.05/27). For other analyses, we considered a two-sided P < 0.05 as statistically significant. All statistical analyses were performed using Stata software version 15.0 (StataCorp., College Station, Texas, USA).

## Supplementary information

Supplementary Figure S1 and Table S1

## Data Availability

The datasets generated during and/or analyzed during the current study are available from the corresponding author on reasonable request.
