# Supplementary material for: Serum amino acid profiles and risk of type 2 diabetes among Japanese adults in the Hitachi Health Study
Source: Sci Rep. 2019 May 7;9:7010. doi: 10.1038/s41598-019-43431-z (PMC6504928; doi:10.1038/s41598-019-43431-z)
Supplement: Supplementary file 1 — Supplementary Figure S1 and Table S1 [file 41598_2019_43431_MOESM1_ESM.pdf]

Supplementary information

Serum amino acid profiles and risk of type 2 diabetes among Japanese adults in the Hitachi Health Study

Sanmei Chen[1,*], Shamima Akter[1], Keisuke Kuwahara[1,3], Yumi Matsushita[2], Tohru Nakagawa[4], Maki Konishi[1], Toru Honda[4], Shuichiro Yamamoto[4], Takeshi Hayashi[4], Mitsuhiko Noda[5], Tetsuya Mizoue[1]

[1]Department of Epidemiology and Prevention, Center for Clinical Sciences, National Center for Global Health and Medicine, Tokyo, Japan

[2]Department of Clinical Research, Center for Clinical Sciences, National Center for Global Health and Medicine, Tokyo, Japan

[3]Teikyo University Graduate School of Public Health, Tokyo, Japan

[4]Hitachi Health Care Center, Hitachi, Ltd., Ibaraki, Japan

[5]Department of Endocrinology and Diabetes, Saitama Medical University, Saitama, Japan

[*]Corresponding author: Sanmei Chen, Department of Epidemiology and Prevention, Center for Clinical Sciences, National Center for Global Health and Medicine, Toyama 1-21-1, Shinjuku Ward, Tokyo 162-8655, Japan. Tel: +81-3-3202-7181, Fax: +81-3-3202-7364, E-mail: sanmei.chen@kyudai.jp

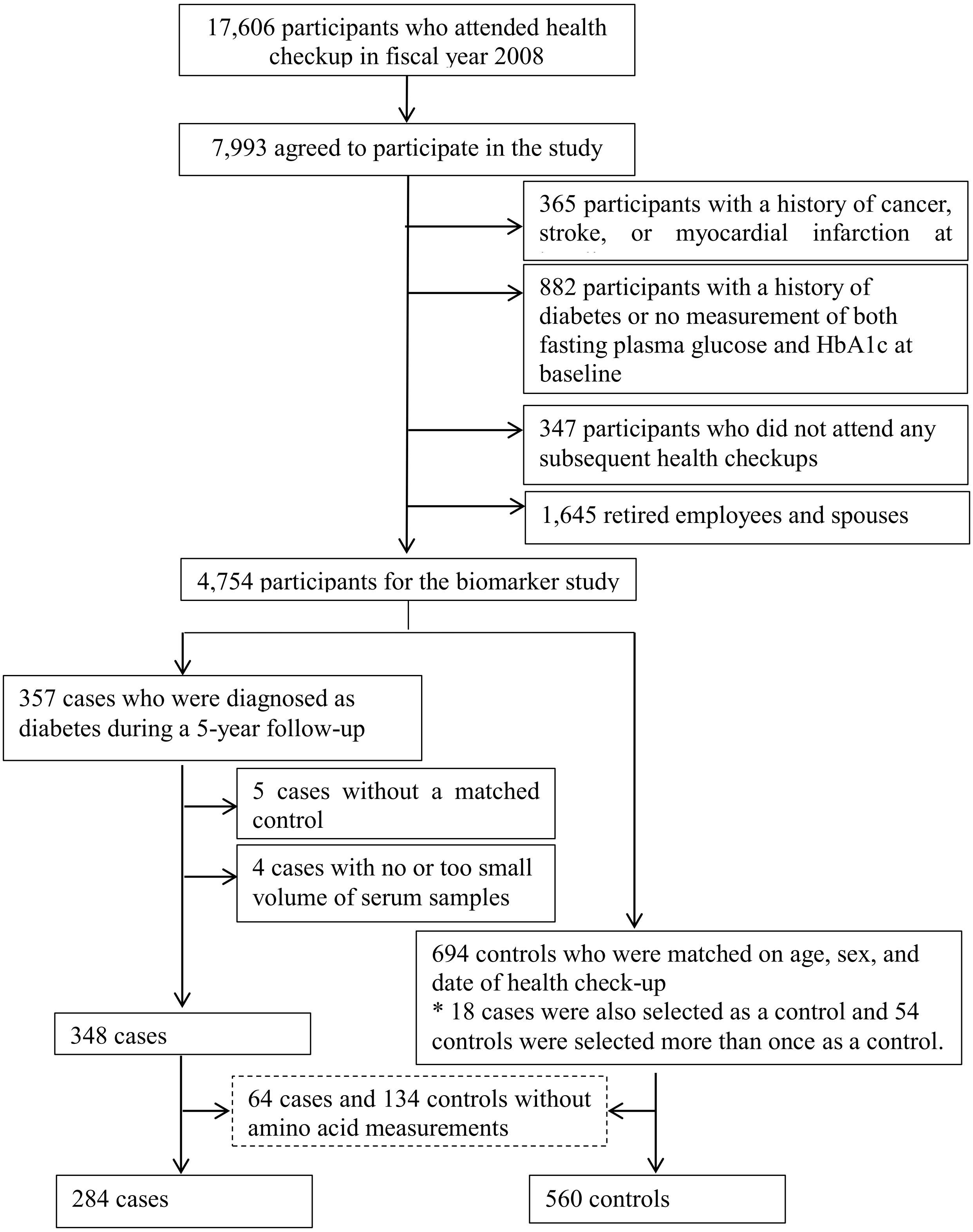

Supplemental Figure S1. Flow diagram of study sample

Supplementary Table S1. Associations of intermediary organic acid concentrations with type 2 diabetes risk in the Hitachi Health Study

	Quartiles (range)[a] (μmol/L)	No. of ca./co.	Model 1 OR (95% CI)	Model 2 OR (95% CI)	Model 3 OR (95% CI)
α-amino-n-butyric acid	1 (< 16.2)	58/136	1.00	1.00	1.00
	2 (16.2 -< 20.4)	80/147	1.27 (0.84-1.92)	1.31 (0.84-2.05)	1.39 (0.86-2.25)
	3 (20.4 -< 24.8)	69/144	1.12 (0.73-1.71)	0.99 (0.62-1.57)	0.88 (0.54-1.45)
	4 (≥ 24.8)	77/133	1.34 (0.88-2.05)	1.04 (0.65-1.66)	1.03 (0.63-1.71)
	P-trend[b]		0.26	0.77	0.63
Hydroxyproline	1 (< 7.6)	61/143	1.00	1.00	1.00
	2 (7.6 -< 9.5)	75/141	1.27 (0.83-1.92)	1.27 (0.81-1.98)	1.24 (0.77-2.00)
	3 (9.5 -< 12.1)	80/135	1.40 (0.93-2.12)	1.22 (0.78-1.92)	1.03 (0.64-1.67)
	4 (≥ 12.1)	68/141	1.17 (0.76-1.80)	1.13 (0.71-1.80)	1.01 (0.61-1.66)
	P-trend[b]		0.57	0.80	0.70
3-methyl-histidine	1 (< 6.6)	69/150	1.00	1.00	1.00
	2 (6.6 -< 7.45)	60/130	1.00 (0.66-1.53)	0.97 (0.61-1.53)	0.93 (0.57-1.52)
	3 (7.45 -< 8.7)	80/149	1.17 (0.79-1.74)	1.22 (0.80-1.87)	1.19 (0.76-1.88)
	4 (≥ 8.7)	75/131	1.25 (0.83-1.88)	1.06 (0.68-1.64)	0.97 (0.60-1.55)
	P-trend[b]		0.22	0.64	0.92
Taurine	1 (< 84.8)	82/140	1.00	1.00	1.00
	2 (84.8 -< 95.6)	61/140	0.75 (0.50-1.12)	0.75 (0.48-1.16)	0.72 (0.45-1.15)
	3 (95.6 -< 107.8)	61/140	0.74 (0.49-1.12)	0.80 (0.51-1.22)	0.83 (0.52-1.32)
	4 (≥107.8)	80/140	0.98 (0.66-1.45)	0.95 (0.62-1.45)	0.87 (0.56-1.36)
	P-trend[b]		0.99	0.91	0.70
Monoethanolamine	1 (< 14.6)	66/136	1.00	1.00	1.00
	2 (14.6 -< 16.1)	61/133	0.95 (0.62-1.45)	0.88 (0.55-1.40)	0.77 (0.47-1.25)
	3 (16.1 -< 18.3)	74/151	1.01 (0.67-1.51)	0.94 (0.61-1.46)	1.04 (0.65-1.65)
	4 (≥ 18.3)	83/140	1.21 (0.81-1.82)	1.08 (0.69-1.68)	1.04 (0.65-1.67)
	P-trend[b]		0.27	0.59	0.59

Abbreviations: OR, odds ratio; CI, confidential interval; BMI, body mass index; HOMA-IR, homeostasis model assessment of insulin resistance.

Model 1 was adjusted for the following matching factors: age (years), sex, and month of health examination (April-June, July-Sep, Oct-Dec, or Jan-March). Model 2 was based on model 1, additionally adjusted for leisure time physical activity (< 150 minutes/week or ≥ 150 minutes/week), occupational physical activity (sedentary or active), smoking (never smoker, former smoker, or current smoker consuming < 20 or ≥ 20 cigarettes/day), alcohol consumption (non-drinker, or alcohol consuming < 23, 23 to < 46, or ≥ 46 g ethanol/day), shift work (yes or no), sleep duration (< 6, 6 to < 7, or ≥ 7 hours/day), family history of diabetes (yes or no), and hypertension (yes or no), and BMI. Model 3 was further adjusted for HOMA-IR.

[a] Quartiles were based on the distribution of serum concentrations among controls.

[b] All linear trend tests over quartiles were conducted by replacing the ordinal values with the median concentration within each quartile.
